# Impact of pulmonary hypertension on neurodevelopmental outcome in preterm infants with bronchopulmonary dysplasia: a cohort study

**DOI:** 10.1038/jp.2016.108

**Published:** 2016-07-21

**Authors:** H Nakanishi, A Uchiyama, S Kusuda

**Affiliations:** 1 Department of Neonatology, Maternal and Perinatal Center, Tokyo Women's Medical University, Tokyo, Japan

## Abstract

**Objectives:**

To evaluate the impact of pulmonary hypertension (PH) on long-term growth and neurodevelopmental outcomes of extremely preterm infants with bronchopulmonary dysplasia (BPD).

**Study Design:**

A single-center retrospective cohort of preterm infants born at <28 weeks gestational age from 2000 to 2011 was evaluated at 3 years of age. Growth and neurodevelopmental outcomes were compared among 3 groups: non-BPD, BPD without PH and BPD with PH. BPD was defined according to oxygen demand at 36 weeks postmenstrual age. PH was diagnosed by echocardiography during the neonatal intensive care unit stay.

**Results:**

Sixty-two infants without BPD, 60 with BPD without PH and 20 with BPD with PH were analyzed. Regardless of PH status, somatic growth was smaller in both BPD groups of infants than in non-BPD infants, with further reduction in the group having BPD with PH. Furthermore, a developmental quotient of <70 was more prevalent in the BPD infants with PH than in the BPD infants without PH (odds ratio (OR): 4.37; 95% confidence interval, CI: 1.16 to 16.5). Multivariate analysis demonstrated that BPD with PH was one of the independent perinatal risk factors for developmental quotient <70 at 3 years of age (OR: 4.94, 95% confidence interval: 1.06 to 24.1).

**Conclusion:**

PH had an additional negative effect on long-term growth and neurodevelopmental outcomes of extremely preterm infants with BPD.

## Introduction

Despite remarkable improvement in neonatal intensive care for preterm infants, bronchopulmonary dysplasia (BPD) remains one of the most significant medical complications associated with higher morbidity and mortality.^1, 2, 3^ Furthermore, the incidence of BPD has not decreased in spite of the improved mortality rate among high-risk infants.^1, 4^ Among many types of cardiorespiratory impairment, pulmonary hypertension (PH) is one of the major complications associated with BPD.^5, 6, 7, 8, 9, 10^ Although most published reports have mentioned the overall incidence of PH in BPD, there have been few reports on the long-term neurodevelopmental outcomes of BPD infants with PH due to the high mortality rate during follow-up periods. However, because of recent advances in the management of PH in the neonatal intensive care units and after discharge, many infants with BPD, even those complicated with PH, are now surviving beyond infancy. Therefore, the aim of the present study was to determine the impact of BPD with or without PH on long-term growth and neurodevelopmental outcomes of preterm infants born at <28 weeks gestational age (GA) and to determine the perinatal risk factors for developing PH.

## Materials and methods

### Study population

**Figure 1:**
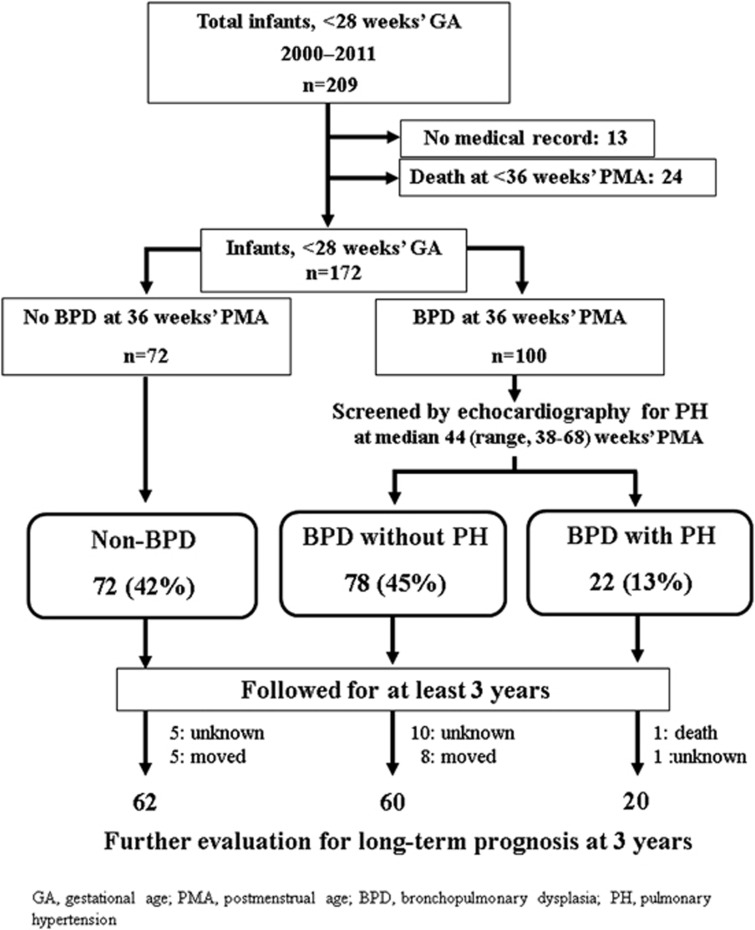
Flow chart of the study infants evaluated. Among 209 infants at <28 weeks GA, 172 were classified into three groups: 72 non-BPD; 78 BPD without PH; 22 BPD with PH. Among those study infants, 142 were followed up until 3 years of age and were evaluated for long-term prognosis. BPD, bronchopulmonary dysplasia; GA, gestational age; PH, pulmonary hypertension.

From January 2000 to December 2011, 209 preterm infants at <28 weeks GA were admitted to the neonatal intensive care units of the Maternal and Perinatal Center, Tokyo Women's Medical University. Among the study infants, there were no life-threatening congenital anomalies or chromosomal anomalies. Thirteen infants were excluded from the study because of incomplete medical records that would hinder evaluation. Twenty-four infants who died before 36 weeks postmenstrual age (PMA) were also excluded. The remaining 172 preterm infants at <28 weeks GA were enrolled in the study and were classified into three groups: non-BPD (*n*=72), BPD without PH (*n*=78) and BPD with PH (*n*=22). Among the 172 study infants, a total of 30 infants were excluded from the evaluation of long-term prognosis at 3 years of age because 13 infants moved to another institution, 16 infants were missed for unknown reasons, and 1 died during hospitalization. Finally, 142 infants [non-BPD (*n*=62), BPD without PH (*n*=60) and BPD with PH (*n*=20)] were evaluated for long-term growth and neurodevelopmental outcomes at 3 years of age (Figure 1).

### Diagnosis of BPD and PH

The diagnosis of BPD was based on the National Institute of Health consensus definition.^11^ Echocardiography was routinely performed by the neonatologists for all preterm infants admitted to the neonatal intensive care units every day until day 7 and as needed thereafter, if clinically indicated. In particular, the infants with BPD underwent screening echocardiography to rule out PH during their neonatal intensive care units stay. The infants without BPD were not subjected to screening echocardiography, unless otherwise clinically indicated. PH was diagnosed by echocardiography if at least one of the following findings was present: (1) right ventricular hypertrophy; (2) flattening of the interventricular septum; (3) velocity of tricuspid regurgitation ⩾3 m s^−1^ in the absence of pulmonary stenosis; and (4) abnormal pulmonary artery Doppler (saw tooth pattern or shortened acceleration time). PH was considered to have resolved if the last echocardiography showed regression of right ventricular hypertrophy, restoration of the interventricular septal configuration, a diminished amount of tricuspid regurgitation or an improved abnormal pulmonary artery flow pattern. Echocardiography was performed before discontinuation of oxygen supplementation therapy; the absence of any clinical symptom, such as tachypnea or tachycardia, was also regarded as an important decision factor for the resolution of PH.

### Definitions

All parameters used in the study were defined according to a registration manual used for the Neonatal Research Network database in Japan.^2, 3, 12^ Briefly, infants with weight and height under the tenth percentile of the normal birth weight and height were defined as small for gestational age (SGA). Clinical chorioamnionitis was diagnosed based on clinical findings. Histologic chorioamnionitis was defined according to the criteria reported by Blanc.^13^ Cerebral palsy was defined at 3 years of age according to the clinical features described by Bax.^14^

### Risk factors

Clinical factors included birth weight, GA, SGA, sex, 1- and 5-min Apgar scores, umbilical cord artery blood gas value on admission to neonatal intensive care units and perinatal characteristics, such as maternal hypertension, maternal/gestational diabetes mellitus, late birth, antenatal steroid use, premature rupture of membranes, clinical chorioamnionitis, histologic chorioamnionitis with Blanc stage ⩾2, umbilical vein immunoglobulin M level >20 mg dl^−1^ and vaginal delivery. Neonatal complications consisted of respiratory distress syndrome, pneumothorax, pulmonary hemorrhage, persistent pulmonary hypertension of the newborn, sepsis, patent ductus arteriosus, intraventricular hemorrhage and necrotizing enterocolitis or gastrointestinal perforation. Factors for estimating short-term prognosis included oxygen supplementation at 36 weeks PMA, postnatal steroid use, day when enteral feeding of 100 ml kg^−1^ per day became established, total duration of mechanical ventilation and hospital stay, home oxygen therapy, retinopathy of prematurity, hearing abnormality and periventricular leukomalacia.

### Long-term growth and neurodevelopmental assessment

Long-term prognosis was evaluated at 3 years of age based on the incidence of cerebral palsy; somatic growth, such as height, body weight and head circumference; and developmental testing according to the Kyoto Scale of Psychological Development (KSPD).^12, 15^ The KSPD consists of the total developmental quotient (DQ) and three subscales of the DQ for posture-motor, cognition-adaptation and language-social skills.^12^ The KSPD was administered by experienced testers who were certified psychologists who were blinded to the perinatal details of the hospital stay. The DQ for each subscale and the total DQ were estimated from the developmental age defined for each task. A DQ score <70 was interpreted as significantly delayed performance.

To elucidate the impact of PH on DQ<70 at 3 years of age, multiple regression analysis was performed using the following factors: (1) GA and SGA, which were regarded as birth factors; (2) sex, which has been reported to influence growth and development;^12, 16^ (3) severe intraventricular hemorrhage and periventricular leukomalacia, which were regarded as extra-cardiopulmonary factors;^17, 18^ and (4) BPD without PH and BPD with PH as cardiopulmonary factors.^19^

### Statistical analysis

Statistical analysis was performed using JMP 11 (SAS Institute, Cary, NC, USA). Data were summarized as mean±s.d. or median and range. The *χ*^2^-test or Fisher's exact test was used to compare categorical variables. All outcome variables with normal distribution were analyzed in a simple comparison using unpaired *t*-test or one-way analysis of variance (ANOVA), with Tukey's *post hoc* test for comparisons with more than two independent groups. Variables with non-normal distribution were analyzed by Wilcoxon rank sum tests or Kruskal–Wallis one-way ANOVA. A two-sided *P*-value <0.05 was considered significant. The odds ratio with 95% confidence interval (95% CI) extended the interpretation of significant test results.

### Study approval

This study protocol was approved by the institutional review board of Tokyo Women's Medical University, with waiver for informed consent.

## Results

### Baseline characteristics

A total of 22 infants were diagnosed with PH at median PMA of 44 weeks (range, 38 to 68 weeks). The incidence of PH, calculated as a factor of total preterm or BPD infants, was 11% among 209 extremely preterm infants and 22% among 100 BPD infants. Conversely, among the infants without BPD, none showed any clinical symptoms or signs of PH during the follow-up period. Seventy-two infants in the non-BPD group, 78 in the BPD without PH group and 22 in the BPD with PH group were analyzed for the study. During hospitalization, 1 infant in the BPD with PH group died and 29 were lost to follow-up. Therefore, 21 patients in the BPD with PH group were discharged and followed-up every 1 month by pediatric clinicians; every 3 to 6 months, they underwent echocardiography that was performed by pediatric cardiologists. Among these patients, 16 were on oxygen and diuretics at the time of discharge; the conditions of all 16 patients finally resolved by 2 years of age. However, the other five infants received vasodilator therapy in addition to oxygen and diuretics at the time of discharge; three of these infants remained on oxygen and vasodilator therapy at more than 3 years of age.

Among 22 PH infants, echocardiographic findings at initial diagnosis were tricuspid regurgitation in 8 (36%), right ventricular hypertrophy in 3 (14%), septal flattening in 7 (32%), elevated right ventricular pressure in 10 (45%) and abnormal flow pattern with or without oxygen supplementation in 20 (91%).

### Perinatal characteristics and incidence of neonatal complications

Regardless of PH status, GA and birth weight were significantly lower in the BPD group than in the non-BPD group (*P*<0.001). Furthermore (Table 1), GA was much lower in those with BPD with PH (*P*=0.02). Compared with the non-BPD group, the BPD with PH group had significantly lower Apgar scores at both 1 min (*P*=0.02) and 5 min (*P*=0.03). There were no significant differences between groups in the incidence of SGA. The BPD with PH group had a significantly higher incidence of premature rupture of membranes (*P*=0.02), histologic chorioamnionitis Blanc stage ⩾2 (*P*=0.009), umbilical vein immunoglobulin M level >20 mg dl^−1^ (*P*=0.01) and persistent pulmonary hypertension of the newborn (*P*<0.001) than the other two groups.

### Short-term prognosis

Severe BPD was more prevalent in the BPD with PH group (*P*<0.001) than in the other two groups. During hospitalization (Table 2), one infant with PH died because of PH crisis induced by respiratory infection. There were no significant differences among the groups in terms of incidence of periventricular leukomalacia, convulsions and hearing abnormality. Retinopathy of prematurity⩾stage 3 was more prevalent in the BPD groups than in the non-BPD group (*P*=0.04), but there was no difference in its incidence between the BPD infants with PH and those without PH. Home oxygen therapy was more prevalent in the BPD with PH group than in the other two groups (*P*<0.001).

### Long-term growth and neurodevelopmental outcomes

Among the 3 groups, the BPD with PH group was most significantly needed home oxygen therapy at 1.5 years of age (*P*<0.001) (Table 3). There was no difference among the groups in the incidence of cerebral palsy, but there were significant differences in body weight (*P*<0.001), height (*P*=0.003) and head circumference (*P*=0.03) at 3 years of age; the values of each of these three parameters decreased in the order corresponding to non-BPD, BPD without PH and BPD with PH. In particular, body weight (*P*=0.04) was much lower in the infants with PH. There was a significant difference among groups in the prevalence of DQ<70 (*P*=0.04), which was more prevalent in the BPD with PH group than in the other two groups (OR: 3.7, 95% CI: 1.04 to 13.3 vs non-BPD) (OR: 4.37, 95% CI: 1.16 to 16.5 vs BPD without PH).

Multiple regression analysis demonstrated that the significant independent risk factors for DQ <70 were periventricular leukomalacia (adjusted OR: 31.0, 95% CI: 2.51 to 764) and BPD with PH (adjusted OR: 4.94, 95% CI: 1.06 to 24.1).

## Discussion

The major finding of our study was that PH status had an additional negative effect on the long-term growth and neurodevelopmental outcomes of extremely preterm infants with BPD. Furthermore, BPD complicated with PH was one of the significant independent risk factors for delayed neurodevelopmental outcome.

The severity of BPD is known to be correlated with abnormal neurodevelopmental outcomes;^20^ therefore, the poor neurodevelopmental outcome of the BPD with PH group in our study might be due to the severity of the BPD. However, univariate subclass analysis demonstrated that even among 21 severe BPD infants who were followed up at 3 years of age, the infants with PH remained to have a significantly higher incidence of DQ<70 than the infants without PH (46 vs 0%, *P*=0.04). This suggested that PH independently had an additional negative effect on long-term neurodevelopmental outcome among preterm infants.

Previous studies have reported only on the overall incidence and short-term prognosis of infants with PH and BPD,^5, 6, 7, 8, 9, 10^with less information about the long-term growth and neurodevelopmental outcomes. Therefore, the strength of the present study was the examination of the long-term outcomes of BPD infants with PH compared with control infants at 3 years of age. In this study population, the follow-up rate was high at 83% (142 of 172 study infants). Furthermore, to our knowledge, the present study was the first to demonstrate an additional negative effect of PH on long-term growth and neurodevelopmental outcomes among infants with BPD.

Among the perinatal factors, intrauterine infection has been known to be a trigger of lung injury, which leads to BPD in premature infants. The present study demonstrated that intrauterine infection, as indicated by histologic chorioamnionitis Blanc stage ⩾2 and umbilical vein immunoglobulin M level >20 mg dl^−1^, additionally influenced the occurrence of PH in BPD infants. Actually, among the pre- and post-natal risk factors for development of lung injury, chorioamnionitis (CAM) was considered to be essential in the evolution of BPD.^21, 22, 23, 24^ An animal model in which BPD was induced by intra-amniotic injection of lipopolysaccharide demonstrated that antenatal inflammation inhibited endothelial cell protein expression of molecules, such as vascular endothelial growth factor, vascular endothelial growth factor receptor-2, endothelial nitric oxide synthase, platelet endothelial cell adhesion molecule-1 and Tie-2 protein; this was followed by vascular remodeling in small pulmonary arteries.^25^ Exposure to antenatal inflammation may not only be correlated with preterm delivery, but may also directly cause vascular remodeling and contribute to the development of PH. Therefore, a therapeutic strategy against intrauterine infection, with respect to preterm delivery, is important in halting the progression of PH. On the other hand, recent cohort studies showed that chorioamnionitis had no impact on the incidence of BPD and could be protective;^26^ therefore, the role of chorioamnionitis in BPD is still a matter of significant debate.

Among the neonatal complications in the present study, persistent pulmonary hypertension of the newborn had a significantly higher incidence in the BPD with PH group (*P*<0.001) than the other two groups. Mourani *et al.*^9^ reported that the early signs of pulmonary vascular disease at 7 days of age were associated with subsequent development of BPD and late PH in preterm infants. The underlying diseases of some persistent pulmonary hypertension of the newborn infants might be functional and/or anatomical pulmonary hypoplasia due to prolonged drainage of lung fluid after premature rupture of membranes. Mostly, in such cases, there is disordered development of pulmonary vasculature that easily leads to pulmonary vascular disease after intensive mechanical ventilation with high mean airway pressure. Therefore, our results seemed to be related to the results of Mourani *et al.*

As shown in other studies, SGA infants were suggested to be at increased risk for severe PH. An animal model of BPD has demonstrated that intrauterine growth restriction may have lasting effects on lung structure and function, together with alveolar and vascular abnormalities, which are related to BPD-associated PH.^27, 28^ However, in the present study, SGA was not correlated with the onset of PH in BPD. This result was in conflict with the results of other several recent studies, which suggested that SGA status was a strong predictor for the development of PH.^7, 8, 9, 29^ Actually, our study population only had 20 (12%) SGA infants out of a total of 172 study infants. The reason for the absence of an association between SGA and BPD-PH in this study is unknown. It might be partly due to racial/ethnic characteristics^30^ or the absence of maternal hypertension in the group of infants with BPD and PH.^31^ However, it is interesting to note that we did find decreased somatic growth in the postnatal period in all BPD infants; this was consistent with other multi-center data showing postnatal growth failure in infants with severe BPD during their initial hospital course.^32^

Our study had some limitations. In the present study, PH diagnosis was basically dependent on echocardiographic findings, which have some limitations in the precise diagnosis of PH. In particular, if significant tricuspid regurgitation was present, estimated systolic pulmonary artery pressure by echocardiography would be relatively sensitive in identifying PH. However, echocardiography cannot directly estimate pulmonary artery resistance; therefore, the absence of a tricuspid regurgitation jet velocity did not always rule out the presence of severe PH. Actually, up to this time, cardiac catheterization has been accepted as the gold standard for diagnosing PH, but this procedure is so invasive that it cannot be easily performed on premature infants. Although this study also evaluated changes in pulmonary artery flow pattern before and after oxygen supplementation in order to avoid overlooking PH, the possibility of underestimation of the true incidence of PH remains.

The mortality rates of the BPD with PH group in our study were better than those of the other published reports in the first year of life.^5, 7, 8^ Although the follow-up rate in our study was high at 83% (142 of 172 study infants), 20% of the study population was lost to follow-up; this may have affected the results of lower mortality rate in the study.

The KSPD is a standard and validated developmental test that is available at all centers participating in the follow-up study of the Neonatal Research Network in Japan but has not been published or standardized in English. Recently, Kono *et al.* assessed and compared the developmental characteristics by KSPD of Japanese very low birthweight infants at 18 months of corrected age with those using the Bayley III, which is globally used to assess the developmental/cognitive function of preterm children in early childhood. They concluded that developmental delay, defined by an overall KSPD DQ of <70, was equivalent to a Bayley III Cog score of <85.^15^ Therefore, the KSPD can be used to assess the outcomes of preterm and very low birthweight infants in the same manner as the Bayley III.

This was a single-center study, which allowed collection of standardized information on all preterm infants (for example, diagnostic criteria of PH, mechanical ventilation management, oxygen saturation target and the follow-up system of preterm infants after hospital discharge). However, different institutions may have different clinical protocols. Our study group comprised mostly Asian population; caution should be exerted in applying these results to other centers with other racial/ethnic groups. Therefore, additional investigation with a larger number of enrolled infants from multiple institutions is warranted.

## Figures and Tables

**Table 1:** Perinatal characteristics and incidence of neonatal complications among infants with or without pulmonary hypertension in bronchopulmonary dysplasia (BPD) compared with infants without BPD

*Characteristic*	*Non-BPD*	*BPD without PH*	*BPD with PH*	P*–value among groups*	P*–value between BPD without and with PH*	*OR*	*95% CI*
	(n=*72)*	(n=*78)*	(n=*22)*			*BPD with PH vs* *without PH*	
*Basic characteristics*							
Birth weight (g), mean (s.d.)	853 (171)	717 (181)^a^	702 (140)^b^	<0.001^c^	0.93	—	—
Gestational age (week), mean (s.d.)	26.3 (1.2)	25.7 (1.4)^a^	24.8 (1.3)^b^^,^^d^	<0.001^c^	0.02	—	—
SGA, *n* (%)	7/72 (10%)	13/76 (17%)	0/22 (0%)	0.07^e^	0.04	—	—
Male gender, *n* (%)	29/72 (40%)	37/78 (47%)	11/22 (50%)	0.59^e^	1.00	1.11	0.43–2.86
Apgar score							
At 1 min, median (range)	4 (1–8)	4 (1–9)	2.5 (1–8)^b^	0.02^f^	0.14	—	—
At 5 min, median (range)	7 (1–9)	6 (1–9)	5.5 (1–9)^b^	0.03^f^	0.27	—	—
Umbilical cord artery pH, mean (s.d.)	7.33 (0.11)	7.33 (0.07)	7.32 (0.10)	0.89^c^	0.93	—	—
Umbilical cord artery BE, mean (s.d.)	−3.9 (4.7)	−4.0 (4.0)	−3.9 (5.5)	0.97^c^	0.99	—	—

*Perinatal characteristics*							
Maternal hypertension, *n* (%)	7/72 (10%)	11/78 (14%)	0/22 (0%)	0.16^e^	0.12	—	—
Maternal DM/GDM, *n* (%)	1/72 (1%)	6/78 (8%)	0/22 (0%)	0.11^g^	0.33	—	—
Maternal age >35 years, *n* (%)	22/72 (31%)	35/78 (45%)	9/22 (41%)	0.19^e^	0.81	0.85	0.33–2.22
PROM, *n* (%)	25/72 (35%)	25/78 (32%)	14/22 (64%)	0.02^e^	0.01	3.71	1.38–9.99
Received antenatal steroid, *n* (%)	40/71 (56%)	46/77 (60%)	16/22 (73%)	0.39^e^	0.32	1.80	0.63–5.10
Clinical CAM, *n* (%)	17/69 (25%)	10/76 (13%)	4/21 (19%)	0.21^e^	0.49	1.55	0.43–5.56
Histological CAM Blanc stage ⩾2, *n* (%)	35/68 (51%)	33/74 (45%)	18/22 (82%)	0.009^e^	0.003	5.59	1.72–18.1
Umbilical vein IgM level >20 mg dl^−1^, *n* (%)	5/65 (8%)	7/66 (11%)	7/22 (32%)	0.01⩽^e^	0.04	3.93	1.20–12.9
Vaginal delivery, *n* (%)	28/72 (39%)	18/78 (23%)	11/22 (50%)	0.02^e^	0.02	3.33	1.24–8.95

*Neonatal complications*							
RDS, *n* (%)	36/72 (50%)	52/78 (67%)	8/22 (36%)	0.02^e^	0.01	0.29	0.11–0.77
Pneumothorax, *n* (%)	2/72 (3%)	5/78 (6%)	1/22 (5%)	0.61^g^	1.00	0.70	0.08–6.28
Pulmonary hemorrhage, *n* (%)	6/72 (8%)	4/78 (5%)	0/22 (0%)	0.38^g^	0.57	—	—
PPHN, *n* (%)	3/72 (4%)	4/78 (5%)	6/22 (27%)	<0.001^e^	0.007	6.94	1.75–27.5
Sepsis, *n* (%)	13/71 (18%)	23/78 (29%)	8/21 (38%)	0.12^e^	0.44	1.47	0.54–4.03
PDA, *n* (%)	41/72 (57%)	50/78 (64%)	16/22 (73%)	0.37^e^	0.61	1.49	0.52–4.25
IVH, *n* (%)	20/72 (28%)	33/78 (42%)	7/22 (32%)	0.17^e^	0.46	0.64	0.23–1.74
Grade ⩾3, *n* (%)	4/72 (6%)	10/78 (13%)	0/22 (0%)	0.09^e^	0.11	—	—
NEC/Gastrointestinal perforation, *n* (%)	0/72 (0%)	0/78 (0%)	3/22 (14%)	0.002^g^	0.01	—	—

Abbreviations: BPD, bronchopulmonary dysplasia; BE, base excess; CAM, chorioamnionitis; CI, confidence interval; DM, diabetes mellitus; GDM, gestational diabetes mellitus; IVH, intraventricular hemorrhage; NEC, necrotizing enterocolitis; OR, odds ratio; PDA, patent ductus arteriosus; PH, pulmonary hypertension; PPHN, persistent pulmonary hypertension of the newborn; PROM, premature rupture of membranes; RDS, respiratory distress syndrome, SD, standard deviation; SGA, small for gestational age.

a *P*<0.05 non-BPD vs BPD without PH.

b *P*<0.05 non-BPD vs BPD with PH.

c Analysis of variance.

d *P*<0.05 BPD without PH vs with PH.

e *χ*2-test.

f Kruskal–Wallis rank test.

g Fisher's exact test.

**Table 2:** Short-term prognosis of infants with or without pulmonary hypertension in bronchopulmonary dysplasia (BPD) compared with those without BPD

*Prognosis*	*Non-BPD*	*BPD without PH*	*BPD with PH*	P*–value among groups*	P*–value between BPD without and with PH*	*OR*	*95% CI*
	(n=*72)*	(n=*78)*	(n=*22)*			*BPD with PH* vs *without PH*	
*Short-term prognosis*							
BPD							
Oxygen at 28 days of age, *n* (%)	62/72 (86%)	78/78 (100%)	22/22 (100%)	<0.001^a^	—	—	—
Oxygen at 36 weeks' PMA, *n* (%)	0/72 (0%)	78/78 (100%)	22/22 (100%)	<0.001^b^	—	—	—
Severity							
Mild, moderate, *n* (%)	62/62 (100%)	65/78 (83%)	7/22 (32%)	<0.001^b^	<0.001	0.09	0.03–0.27
Severe, *n* (%)	0/62 (0%)	13/78 (17%)	15/22 (68%)	<0.001^b^	<0.001	10.70	3.65–31.4
O_2_ concentration at 36 weeks' PMA, mean (s.d.)	21.0 (0.0)	25.3 (3.0)^c^	34.4 (11.3)^d^^,^^e^	<0.001^f^	<0.001	—	—
Steroid treatment for BPD, *n* (%)	9/72 (13%)	47/78 (60%)	20/22 (91%)	<0.001^b^	0.009	6.60	1.44–30.2
Days of ventilation, median (range)	29.5 (0–77)	52 (0–103)^c^	70 (25–173)^d^^,^^e^	<0.001^g^	0.012	—	—
Days when enteral feeding of 100 mL kg^–1^ per day became established, median (range)	19 (10–102)	21 (9–81)	24 (10–132)	0.42^g^	0.82	—	—
Hospital stay, median (range)	124.5 (89–229)	147 (99–330)^c^	170 (119–406)^d^^,^^e^	<0.001^g^	0.003	—	—
PVL, *n* (%)	4/72 (6%)	4/78 (5%)	0/22 (0%)	0.78^a^	0.57	—	—
ROP ⩾Stage 3, *n* (%)	9/70 (13%)	22/78 (28%)	7/22 (32%)	0.04^b^	0.79	1.19	0.43–3.30
Convulsion, *n* (%)	2/72 (3%)	2/77 (3%)	1/22 (5%)	0.69^a^	0.53	1.79	0.15–20.7
Hearing abnormality, *n* (%)	1/67 (1%)	3/77 (4%)	1/20 (5%)	0.54^a^	1.00	1.30	0.13–13.2
HOT at discharge, *n* (%)	0/72 (0%)	4/78 (5%)	21/21 (100%)	<0.001^b^	<0.001	—	—
Death after 36 weeks' PMA, *n* (%)	0/72 (0%)	0/78 (0%)	1/22 (5%)	0.13^a^	0.22	—	—

Abbreviations: BPD, bronchopulmonary dysplasia; CI, confidence interval; HOT, home oxygen therapy; OR, odds ratio; PH, pulmonary hypertension; PMA, postmenstrual age; PVL, periventricular leukomalacia; ROP, retinopathy of prematurity; SD, standard deviation.

a Fisher's exact test.

b *χ*2-test.

c *P*<0.05 non-BPD vs BPD without PH.

d *P*<0.05 non-BPD vs BPD with PH.

e *P*<0.05 BPD without PH vs with PH.

f Analysis of variance (ANOVA).

g Kruskal–Wallis rank test.

**Table 3:** Long-term prognosis of infants with or without pulmonary hypertension in bronchopulmonary dysplasia (BPD) compared with those without BPD

*Prognosis*	*Non-BPD*	*BPD without PH*	*BPD with PH*	P*-value among groups*	P*-value between BPD without and with PH*	*OR*	*95% CI*
	(n=*62)*	(n=*60)*	(n=*20)*			*BPD with PH* vs *without PH*	
*Long-term prognosis*							
CP at 3 years, *n* (%)	6/62 (10%)	2/60 (3%)	0/20 (0%)	0.26^a^	0.41	—	—
HOT at 1.5 years, *n* (%)	0/62 (0%)	0/60 (0%)	7/20 (35%)	<0.001^a^	<0.001	—	—

*Growth at 3 years*							
Body weight (kg), mean (SD)	12.4 (1.6)	11.8 (1.9)	10.7 (1.3)^b^^,^^c^	<0.001^d^	0.04	—	—
Height (cm), mean (s.d.)	89.7 (3.2)	88.0 (4.0)^e^	86.6 (2.9)^b^	0.003^d^	0.31	—	—
Head circumference (cm), mean (s.d.)	48.3 (2.0)	47.9 (2.0)	47.2 (1.1)^c^	0.03^f^	0.35	—	—

*Cognitive development*							
KSPD DQ in all areas <70, *n* (%)	6/58 (10%)	5/56 (9%)	6/20 (30%)	0.04^g^	0.03	4.37	1.16–16.5
All areas, mean (s.d.)	90 (20)	93 (15)	88 (23)	0.54^d^	0.54	—	—
Postural–motor, mean (s.d.)	97 (23)	98 (18)	89 (25)	0.27^d^	0.24	—	—
Cognitive–adaptive, mean (s.d.)	90 (20)	92 (15)	87 (21)	0.59^d^	0.57	—	—
Language–social, mean (s.d.)	91 (22)	92 (19)	88 (28)	0.84^d^	0.82	—	—

Abbreviations: BPD, bronchopulmonary dysplasia; CI, confidence interval; CP, cerebral palsy; DQ, developmental quotient; HOT, home oxygen therapy; KSPD, Kyoto Scale of Psychological Development; OR, odds ratio; PH, pulmonary hypertension; SD, standard deviation.

a Fisher's exact test.

b *P*<0.05 non-BPD vs BPD with PH.

c *P*<0.05 BPD without PH vs with PH.

d Analysis of variance (ANOVA).

e *P*<0.05 non-BPD vs BPD without PH.

f Kruskal–Wallis rank test.

g *χ*2-test.
